# Breaking the boundaries: the power of ligatures in visual-word recognition

**DOI:** 10.3389/fpsyg.2023.1166192

**Published:** 2023-06-13

**Authors:** María Fernández-López, Manuel Perea, Ana Marcet

**Affiliations:** ^1^Department of Methodology of Behavioral Sciences and ERI-Lectura, Universitat de València, Valencia, Spain; ^2^Center of Research in Cognition, Universidad Antonio de Nebrija, Madrid, Spain; ^3^Grupo de Investigación en Enseñanza de Lenguas (GIEL), Department of Language and Literature Teaching, Universitat de València, Valencia, Spain

**Keywords:** word recognition, lexical access, reading, lexical decision, visual similarity

## Abstract

**Introduction:**

Current neurobiological-inspired models of visual-word recognition propose that letter detectors in the word recognition system can tolerate some variations in the visual form of the letters. However, it is unclear whether this tolerance extends to novel ligatures, which combine two letters into a single glyph.

**Methods:**

To investigate this, the present study utilized a masked priming experiment with a lexical decision task to examine whether primes containing novel ligatures are effective in activating their corresponding base word, relative to omitted-letter primes, in the initial stages of word processing. For each target word (e.g., VIRTUAL), were created an identity prime (virtual), a prime containing a novel ligature of two of the letters (e.g., virtual; “ir” in a single glyph), and an omitted-letter prime where one letter was removed (e.g., vrtual [omitted-vowel] in Experiment 1; vitual [omitted-consonant] in Experiment 2).

**Results:**

Results showed that the presence of a novel ligature in the prime resulted in faster lexical decision times compared to a prime with an omitted vowel (Experiment 1), but not with an omitted consonant (Experiment 2). Furthermore, the performance with the primes containing the novel ligature was not different from that of the identity primes.

**Discussion:**

These results suggest that the word recognition system can quickly enable separate letter detectors for novel ligatures. These findings have important implications for our understanding of the front-end of visual-word recognition.

## 1. Introduction

One of the most persuasive demonstrations of our proficiency in reading is our ability to effectively read CAPTCHAs with ease (Hannagan et al., 2012) as well as words written in a style of poor penmanship (Barnhart and Goldinger, 2010). According to the Local Combination Detector (LCD) model of visual-word recognition (Dehaene et al., 2005), this is due to the presence of hierarchical layers of neurons that form the pathway between the visual cortex and the brain regions responsible for encoding word forms. These layers process visual information, from the simplest visual features to the abstract representations of letters and words. Critically, the layers dedicated to letter recognition tolerate a certain level of “shape distortion” (Dehaene and Cohen, 2007, p. 456; see also Grainger and Dufau, 2012; Vergara-Martínez et al., 2021; Fernández-López et al., 2023), allowing us to identify distorted letters in words like TABLE (see also Norris and Kinoshita, 2012, for similar ideas regarding the existence of a noisy-channel during letter and word encoding).

A consequence of the above assumption is that the encoding of letter identity should be subject to some uncertainty in the early stages of visual word recognition, thus resulting in visual similarity effects. To examine this issue, researchers have often employed the masked priming paradigm in conjunction with a lexical decision task (Forster and Davis, 1984). For instance, Perea et al. (2008) found that a target word such as MATERIAL elicited faster responses when the replaced-letter prime was composed of visually similar digits, referred to as “leet” primes (e.g., M4T3R14L, with 4 replacing A), compared to a control prime like M6T2R76L (see Molinaro et al., 2010 for evidence using event-related potentials). The response times for the “leet” prime were similar to those for the identity prime. This pattern was also observed when visually similar letters were used to replace original letters. Marcet and Perea (2017) found that responses to a target word such as OBJECT were faster when the prime was created by replacing a letter with a visually similar one (e.g., obiect) compared to a visually dissimilar prime (obaect), with response times for the visually similar prime comparable to those elicited by the identity prime (obiect ≈ object) (see Gutiérrez-Sigut et al., 2019, for a replication using event-related potentials; see Marcet and Perea, 2018b, for evidence of these effects in the parafovea during sentence reading; see Lally and Rastle, 2023, for evidence with a forced-choice identification task).

One relevant question in this context is whether the ideas of tolerance to shape distortion also affect pairs of letters that resemble a single letter, such as rn (e.g., in the docurnent), may enable not only the detectors for the letters r and n, but also the detector for the letter m. Marcet and Perea (2018a) examined this issue in a masked priming lexical decision task and found that, for the word DOCUMENT, the visually dissimilar prime docurnent was roughly as effective as the identity prime (i.e., document) and more effective than the control prime docusnent. Therefore, it is not only the case that letter detectors during word recognition are facilitated by visually similar digits or letters (such as “4” resembling “A” or “i” resembling “j”), but also that these effects can extend across letter boundaries (for example, rn resembling m).

The goal of the current masked priming experiments was to investigate a complementary and potentially more challenging scenario for models: whether an item like virtual, which has a novel ligature (i.e., ir, where the letters “i” and “r” are joined into a single glyph), enables the encoding of one only of its two letters (“i” or “r”) or the two (“i” and “r”). Would the word recognition system activate only one letter per glyph, as in in slot-channel orthographic schemes (McClelland and Rumelhart, 1981), or would the letter detectors activate in parallel two different letter detectors for the same glyph? It is noteworthy that we often encounter ligatures in words without even noticing it. This is the case, in some fonts, for some of the letters that follow the letter “f” (e.g., the words final and final). Ligatures are also commonly employed in logotypes, as in the one from Banĸia, a former Spanish bank, which had a ligature combining the letters “n” and “k.” Another instance is the German monitor manufacturer, Belinea, which served as the fortuitous logotype that aided in the conception of the present experiment.

Thus, the theoretical issue in the current study is whether, during word recognition, a novel ligature (e.g., ir) activates two separate letter detectors (i.e., the detectors for the two constituent letters) or whether it only activates one letter detector (e.g., either the dot or the curve in “ir” would be disregarded as noise). The experiment was designed following the same methodology as the lexical decision experiments conducted by Marcet and Perea (2017, 2018a). That is, the relationship between an uppercase target and a lowercase prime was manipulated. In Experiment 1, for a target word like VIRTUAL, always containing the letter “i” in an internal position, three primes were created: (1) an identity prime (virtual); (2) a ligature-prime, in which the vowel “i” and a consonant starting with a vertical bar were combined (virtual); and (3) an omitted-vowel prime, in which the vowel “i” from the ligature was removed (vrtual) We prefer to introduce the logic of Experiment 2 later.

Experiment 1 formulated several predictions based on the assumption that letter detectors in the word recognition system are resilient to variations in the visual form of letters (e.g., LCD model: Dehaene et al., 2005). First, it was hypothesized that a prime containing a ligature of the letters “i” and “r” (e.g., virtual) would enable the activation of these letter detectors, resulting in better processing of the target word VIRTUAL than an omitted-vowel prime (e.g., vrtual) in which the letter detector for “r” but not for “i” would be activated. Furthermore, the similar prime containing the ligature (e.g., virtual) could potentially be nearly as effective as an identity prime based on previous research on visual similarity. This finding would be consistent with the view that the word identification system treats novel ligatures as two separate letters. Alternatively, a more rigid slot-coding perspective (McClelland and Rumelhart, 1981; Grainger and Jacobs, 1996; see also Davis, 2010) would predict that each separate glyph only activates one letter detector (i.e., would activate the detectors of either “i” or “r”). Therefore, for the target word VIRTUAL, the ligature prime would not be more effective than the omitted-vowel prime (i.e., they would differ from one letter with the target word) and would be less effective compared to the identity prime. The results suggest limitations to the flexibility of the orthographic scheme within a glyph when reading, and challenge the assumption of resilient letter detectors in Dehaene et al.’s (2005) LCD model.

## 2. Experiment 1

### 2.1. Method

#### 2.1.1. Participants

We recruited thirty native speakers of Spanish, all of them undergraduate psychology students at the University of València (27 women). This sample size, the same as Marcet and Perea’s (2018a) Experiment 1, was chosen to have 2,400 observations per condition, thus above the minimal recommendations suggested by Brysbaert and Stevens (2018). The participants had normal (corrected) vision of the participants and no record of reading difficulties. All participants signed a written consent form and the experiment was approved by the Ethics Committee of Experimental Research of the University of València.

#### 2.1.2. Procedure

The study was conducted in small groups of three to four individuals within a quiet laboratory setting. The experiment was implemented utilizing the DMDX software (Forster and Forster, 2003) on Windows-based computers. Each trial began with the presentation of a series of hash marks at the center of the computer screen for a duration of 500 milliseconds, followed by the presentation of a lowercase prime for 50 milliseconds, and finally, an uppercase target stimulus. The target remained on the screen until either the participant provided a response or the 2000-millisecond deadline had elapsed, at which point the response was recorded as an error. Participants were instructed to determine whether a presented letter string constituted a word by pressing either a green key (indicating “yes”) or a red key (indicating “no”). They were advised to make their decisions as quickly as possible while maintaining a high degree of accuracy. The experiment, consisting of 240 word trials and 240 nonword trials, lasted approximately 25 min in total. Prior to the experimental trials, a 16-trial practice phase was conducted, and three breaks were integrated into the course of the experiment.

#### 2.1.3. Materials

In order to serve as target words, we selected 240 Spanish words, each containing five to nine letters (with a mean length of 7.5 letters), from the Spanish database (Duchon et al., 2013). The average frequency per million in the Espal subtitle database was 22.1 (ranging from 0.2 to 235.5). Each word contained an internal sequence of letters, either “ir” (e.g., virtual), “in” (e.g., singular), or “im” (e.g., similar). For each target word, which was always displayed in uppercase letters (e.g., VIRTUAL), we generated three primes, all displayed in lowercase: (1) an identity prime that was nominally identical to the target (e.g., virtual); (2) a ligature-prime in which the ir/in/im sequence was replaced with a ligature of the corresponding letters (e.g., virtual); and (3) an omitted-vowel prime in which the letter “i” from the ligature was deleted (e.g., vrtual). To create the glyphs with the ligatures, we employed a font editor, TypeLight (CR8 Software Solutions Ltd, 2020). Additionally, we created 240 orthographically legal nonwords using Wuggy (Keuleers and Brysbaert, 2010), ensuring that they contained the same ir/in/im combinations as the target words (e.g., dimalar, vinitre, dirtuar). The prime-target manipulation for nonword targets was equivalent to that of word targets. To balance the prime-target combinations, we created three lists in a Latin Square design. For example, the target word VIRTUAL could be preceded by virtual in List 1, virtual in List 2, and vrtual in List 3, and all participants would receive 80 items per condition (80 * 3 = 240 word targets, 80 * 3 = 240 nonword targets). The set of stimuli is available in the link indicated in the Data Availability section.

### 2.2. Results and discussion

As is the norm in word recognition experiments, latencies associated with very brief response times (RTs) (<250 ms; 4 trials [less than 0.03% of trials]) and error responses (6.0% for words and 6.7% for nonwords) were excluded from the latency analysis. Table 1 presents the mean lexical decision times and accuracy for the three prime-target conditions, both for words and nonwords. Our primary focus was on the word data, as masked priming effects in nonword targets are typically minimal and unreliable.

**Table 1:** Mean response times (in ms) and error rate (in percentages) in the prime-target conditions for the stimuli in Experiment 1.

	Identity	Ligature	Omitted vowel
Words	606 (6.2)	600 (5.8)	610 (6.0)
Non-words	702 (6.5)	710 (6.8)	709 (6.9)

The statistical analysis followed the same method used in the Marcet and Perea (2018a) experiment. We utilized (generalized) linear mixed-effects models, implemented through the lme4 package (Bates et al., 2015) in the R environment (R Core Team, 2022), to conduct inferential analysis. The lmerTest package (Kuznetsova et al., 2017) was used to obtain *p* values. To reduce the positive skewness of the RT distribution in the linear mixed-effect model of the latency data employed an inverse-1,000/RT transformation, thus reflecting the number of words per second—note that the negative sign was added to keep the same direction of the effects as in the raw data. We chose the binomial distribution to fit the generalized linear mixed-effect model of the accuracy data. The only fixed factor in the model was Prime Type (identity, ligature, omitted-vowel), with ligature prime as the reference level, thereby allowing us to compare the omitted-vowel prime with it, and also test the effectiveness of the ligature prime compared to the identity prime. We included the most complex model of intercepts and slopes for subjects and intercepts that converged—this was a model including slopes for both subjects and items. For completeness, parallel Bayesian Linear Mixed-Effects Models were run using the brms package (Bürkner, 2016) with the maximal random-effect structure (1 + PrimeType|subject) + (1+ PrimeType|item). These analyses produced the same results as reported here (see the Data Availability section for a link to the scripts and outputs).

*Ligature* vs. *Omitted-Vowel*. We found faster responses to the target words (around 10 ms) when preceded by the ligature prime than when preceded by the omitted-vowel prime, *b* = 0.026, *SE* = 0.009, *t* = 2.77, *p* = 0.006. There were no differences between these two conditions in the accuracy data (*t* < 1, *p* > 0.70).

*Identity* vs. *Ligature*. We did not find any significant differences between the ligature and identity priming conditions in the latency data (*t* = 1.37, *p* = 0.17) or accuracy data (*t* < 1, *p* > 0.50).

Thus, the primes composed of novel ligatures (e.g., virtual) were more effective than omitted-vowel primes in which the letter “i” was removed (e.g., vrtual). To examine whether this different was shaped by the type of ligature, we carried out an exploratory analysis computing the median difference between the omitted-vowel and ligature priming conditions across the three letter combinations used in the experiment. The difference was large for the ligatures with “ir” (21 ms), still sizeable for the ligatures with “in” (13 ms), and negligible for the ligatures with “im” (−1 ms). An explanation for this is that the larger the space occupied by the second glyph in the ligature, the more chances that the second (larger) constituent would be the only one enabling a letter detector (i.e., a minimal effect for “im”, an intermediate effect for “in” large effect for “ir”).

As suggested by the Reviewers, the dot on the letter “i” in the ligature prime might enable the detector of the letter “i” over the consonant, especially when the accompanying letter was small-sized (i.e., ir and in). The logic is that the dot is a very distinctive feature that may make the prime virtual to be processed as vitual (see Perea et al., 2021). If this is the case, for the target VIRTUAL, the ligature prime would be processed as vitual, disregarding the consonant. To examine this possibility, we conducted a new experiment in which the omitted-letter condition consists of primes that retain the letter “i” while excluding the consonant (e.g., vitual). If we find differences between the omitted-consonant and ligature primes, it would offer conclusive evidence that the ligature activates both letters. Alternatively, if no differences are observed between the effectiveness of vitual and virtual, it would suggest that the ligature enables mainly the detector of the letter “i.”

## 3. Experiment 2

### 3.1. Method

#### 3.1.1. Participants

We recruited twenty participants from the same population as in Experiment 1.

#### 3.1.2. Materials and procedure

They were the same as in Experiment 1 except that that we only employed two priming conditions: ligature vs. omitted-consonant primes (e.g., virtual, vitual). We also excluded ten target words in which the omitted-consonant prime created a Spanish word (e.g., propia-PROPINA [own-TIP]). We excluded ten target nonwords to keep a 50% proportion of words/nonwords.

### 3.2. Results and discussion

The statistical analyses paralleled those reported in Experiment 1, except that we only included the ligature versus omitted-letter primes—no RTs were faster than the 250-ms cutoff. Table 2 presents the averages per condition.

**Table 2:** Mean response times (in ms) and error rate (in percentages) in the prime-target conditions for the stimuli in Experiment 2.

	Ligature	Omitted consonant
Words	596 (4.1)	599 (4.1)
Non-words	684 (3.7)	682 (3.2)

In the RT analysis for words, we found a non-significant 3-ms advantage of the ligature primes (*b* = 0.008, *SE* = 0.008, *t* = 0.92, *p* = 0.36). We found no signs of an effect in the accuracy analysis (*t* < 0.10, *p* > 0.90).

The negligible 3-ms advantage of the prime virtual over the prime vitual favours the idea that ligature primes activate primarily the letter “i” rather than the two constituent letters. For completeness, similar to Experiment 1, we tested whether some ligature combinations were more effective than others. This exploratory analysis showed a minimal advantage for the ligature primes containing “ir” (4.5 ms) and “in” (4.25 ms), and a small disadvantage for the ligatures with “im” (−6.1 ms). As in Experiment 1, this pattern suggests that ligature primes could be less effective when their constituent glyphs occupy an ample space.

## 4. General discussion

We conducted two masked priming experiments to analyze whether primes containing a glyph with a novel ligature such as “ir” in the prime virtual are more effective at facilitating the recognition of a target word like VIRTUAL than omitted-vowel primes like virtual (Experiment 1) or omitted-consonant primes like vitual (Experiment 2). Experiment 1 revealed that the primes containing a novel ligature were effective at activating their corresponding target words when compared to identity primes (see Marcet et al., 2020; see also Perea et al., 2022, for similar outcomes). More important, Experiment 1 also revealed a processing advantage of ligature-based primes over the primes with an omitted vowel. The magnitude of this difference was approximately equivalent to that reported in prior masked priming experiments that examined visual similarity effects (e.g., obiect-OBJECT vs. obaect-OBJECT; Marcet and Perea, 2017, 2018a). Experiment 2 tested whether the advantage of the ligature prime over the omitted-vowel prime could have been due to the informativeness of the vowel “i” in the ligature prime. To that end, we compared the ligature primes and omitted-consonant primes (e.g., vitual). In this case, we only found a non-significant 3-ms advantage for the ligature primes, thus suggesting that the novel ligature enabled the letter detectors of the salient letter “i” rather than its accompanying consonant.

Thus, the findings of the present experiments, introducing a novel manipulation with ligatures, strongly suggest that the word recognition system is extremely flexible at extracting letter identities from printed words. Specifically, a novel glyph such as “ir” can enable the letter detectors of at least one of its constituent letters (in particular, the letter detector of “i”). Notably, the exploratory analyses in both experiments suggested that letter identity encoding in this scenario could be shaped by the space occupied by the letters in the ligature (e.g., im appears to be less effective that in). This result constrains the front-end of orthographic coding scheme in computational models of word recognition, as they are not fully dependent on the number of slots (or glyphs), but rather on the information provided by each of the glyphs. Furthermore, it is important to consider the present results in the context of a complementary finding that revealed that letter combinations (e.g., rn in docurnent) can activate single letter detectors (e.g., rn can activate m in masked priming; see Marcet and Perea, 2018a). Taken together, these findings suggest that there is a processing window in which the word identification system has some uncertainty not only on the specific letter identities but also on the nature and number of the potential letter identities (see Norris and Kinoshita, 2012; Marcet and Perea, 2018a).

**Figure 1:**
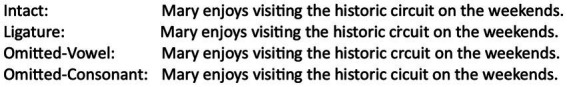
Example of sentence containing a ligature-based word, an omitted-vowel word, and an omitted-consonant word.

To sum up, the present masked priming experiments provide evidence that the word recognition system is capable of extracting letter information from novel ligatures. These results reveal that the front-end of the word recognition system is not solely dependent on the number of glyphs, but rather on the information provided by each of the constituent letters, thus constraining the implementation of models of visual-word recognition. Further research is necessary to determine the underlying mechanisms driving this effect (e.g., via recording the participants’ eye movements during the reading of sentences containing intact vs. ligature-based words; see Figure 1).

## Data availability statement

The datasets presented in this study can be found in online repositories. The names of the repository/repositories and accession number(s) can be found below: The datasets, scripts, and outputs for this study can be found in the following OSF link: https://osf.io/c5msy/?view_only=01d1dc873d2541cf9c6aa2940a5fb5f7.

## Ethics statement

The studies involving human participants were reviewed and approved by the Ethics Committee of Experimental Research of the University of Valencia (#1894511). Written informed consent to participate in this study was provided by the participants.

## Author contributions

AM, MP, and MF-L contributed to the initial conception and design of the study. AM conducted the experiments and wrote some sections. MF-L and MP performed the statistical analyses and wrote the first draft of the manuscript. All authors contributed to the article and approved the submitted version.

## Funding

This study was supported by the Spanish Ministry of Science and Innovation (PID2020-116740GB-I00 funded by the MCIN/AEI/10.13039/501100011033, and PRE2018-083922) and by the Department of Innovation, Universities, Science, and Digital Society of the Valencian Government (CIAICO/2021/172).

## Conflict of interest

The authors declare that the research was conducted in the absence of any commercial or financial relationships that could be construed as a potential conflict of interest.

## Publisher’s note

All claims expressed in this article are solely those of the authors and do not necessarily represent those of their affiliated organizations, or those of the publisher, the editors and the reviewers. Any product that may be evaluated in this article, or claim that may be made by its manufacturer, is not guaranteed or endorsed by the publisher.
